# GOLDEN fusion: a graph-oriented learning with domain-embedding network fusion for generating super gene sets in functional genomics

**DOI:** 10.1093/bib/bbag244

**Published:** 2026-05-25

**Authors:** Qi Li, Cody Nichols, Robert S Welner, Jake Y Chen, Wei-Shinn Ku, Zongliang Yue

**Affiliations:** Mathematics and Computer Science Department, School of Natural Sciences Mathematics & Business, Fisk University, 1000 17th Ave N, Nashville, TN 37208, United States; Computer Science and Software Engineering Department, Samuel Ginn College of Engineering, Auburn University, 3101 Shelby Center for Engineering Technology, Auburn, AL 36849, United States; Hematology & Oncology, Heersink School of Medicine, University of Alabama at Birmingham, 1720 2nd Avenue South, Birmingham, AL 35233, United States; Biomedical Informatics and Data Science, Heersink School of Medicine, University of Alabama at Birmingham, 1720 2nd Avenue South, Birmingham, AL 35294, United States; Computer Science and Software Engineering Department, Samuel Ginn College of Engineering, Auburn University, 3101 Shelby Center for Engineering Technology, Auburn, AL 36849, United States; Department of Health Outcomes Research and Policy, Harrison College of Pharmacy, Auburn University, 2316 Walker Building, Auburn, AL 36849, United States

**Keywords:** PAGER, connection-based embedding, semantic-based embedding, functional genomics, integrative biology, LLM

## Abstract

The integrative analysis of gene sets, networks, and pathways is pivotal for deciphering omics data in translational biomedical research. To significantly increase gene coverage and enhance the utility of gene sets from diverse sources, we introduced pathways, annotated gene lists, and gene signatures (PAGs) enriched with metadata to represent biological functions. Furthermore, we established PAG–PAG networks by leveraging gene member similarity and gene regulations. However, in practice, high similarity in descriptions and gene membership often produces redundant, lengthy PAG lists, leading to gene set enrichment results that are difficult to interpret. We present Graph-Oriented Learning with Domain-Embedding Network (GOLDEN) fusion, an integrative framework that jointly leverages (i) connection-based embeddings derived from PAG–PAG relationships and (ii) semantic-based embeddings learned from PAG descriptions with a large language model (LLM). The two representations are combined via early fusion with a tunable weighting to produce a unified embedding on which clustering identifies concise, higher-level super-PAG. To assess when clustering is appropriate, we introduce a Connection Disparity Index, trained on synthetic stochastic block models, as a proxy for network “clusterability.” We further optimize the number of clusters with consensus clustering. On Gene Ontology Annotation biological process benchmarks, GOLDEN fusion recovers *is-a* structure more accurately than either connection-only or semantic-only baselines, demonstrating consistent gains in Adjusted Rand Index and Normalized Mutual Information. Finally, we generate summaries for each super-PAG by synthesizing its member PAG descriptions using a comparative analysis of LLMs. GOLDEN fusion provides an integrated framework for interpreting omics results.

## Introduction

Systems biology, when coupled with high-throughput omics technologies like proteomics, allows for the discovery of novel pathway biomarkers, which are essential for understanding complex diseases [1]. Functional genomics analysis and systems biology are critical and fundamental for understanding the genetic basis of human health [2, 3]. Previous work has demonstrated how coupled systems biology and proteomics methods can reveal pathway-level biomarkers crucial for exploring complex disease mechanisms [1]. It enhances our knowledge of pathogenic pathways by examining the interactions between different types of molecules, ultimately creating a comprehensive genotype–phenotype map [4]. Early work in signaling network systems biology established the groundwork for these integrative approaches [5]. With a deeper understanding of the molecular processes driven by complex molecular interactions and chemical reactions, biomedical discovery is expedited, leading to numerous applications in the study of complex diseases. These include molecular risk stratification [6–8], preclinical drug screening [9–11], prognosis prediction [12–14], new biomarker discovery [15–17], and precision medicine [18–20]. With the advent of new and advanced techniques for multi-omics high-throughput sequencing, a vast amount of genomics data has been generated, presenting both challenges and opportunities. This data illuminates the complex interactions among genes, gene products, and the environment, ushering in a new era of molecular understanding and leading to novel hypotheses regarding the prevention and treatment of complex diseases. Integrative systems biology approaches, combined with data mining techniques, have been crucial for uncovering the role of genetic variations in diseases and mining disease-specific molecular association profiles across complex datasets [21–25]. Complementary datasets like HIP2 provide foundational plasma proteomics data for benchmarking and network integration [26]. Network-based systems biology approaches have been instrumental in constructing pathway-connected networks, analyzing dose–response relationships, and prioritizing drug targets for repositioning in various diseases, from infectious diseases to cancer [27–29].

Computational functional genomics leverages computational approaches to address challenges in functional genomics, enhancing data interpretability and facilitating novel knowledge discovery. For a deeper understanding of molecular pathways and genetic control mechanisms [30], various tools and databases have been developed to perform gene set enrichment analysis by processing a gene list to extract a statistical measure of shared biological features. These include Gorilla [31], DAVID [32], WebGestalt [33], EnrichR [34], PANTHER Gene List Analysis [35], TermMapper [36], and g:profiler [37], which are frequently used to provide valuable interpretations into high-throughput omics. With increasing demands from multi-omics studies, several challenges arise. These include adequately covering the extensive content of multi-omics data, rendering complex network-based models, and providing advanced features for in-depth insights in integrative analysis [38]. These network-based approaches have been successfully applied to biomarker discovery and subtyping in cancer, such as breast cancer plasma protein studies [39]. Therefore, the integrative gene sets, networks, and pathways analysis (GNPA) has been introduced and leads the new height of information integration in the recently developed GNPA application [40], such as PyGNA [41], NDEx IQuery [42], PAGER web APP [38], and PANGEA [43]. Meanwhile, the incline of heterogeneous resources has significantly enhanced the analytical power, enabling comprehensive analysis from various sources, including pathways (Reactome [44], KEGG [45], BioCarta, WikiPathways [46], DMaps [47], SPIKE, NCI Nature Curated pathway [48], and GeoMx Cancer Transcriptome Atlas [49]), ontology annotations (Gene Ontology Annotation [50] also known as GOA and Human Phenotype Ontology), tissue-specific expressions (GTEx [51], NGS Catalog [52], and TCGA [53], PathologyAtlas [54], and FANTOM5 [55]), cell-specific expressions (CellAtlas [56]), disease phenotypes (GAD, GWAS Catalog [57], Phewas [58], and Online Mendelian Inheritance in Man), drug–targets (PharmGKB [59] and DSigDB [60]), and miRNA–gene interactions (Microcosm Targets, TargetScan [61], and mirTARbase [62]). Pathways, annotated gene lists, and gene signatures (PAGs) have been introduced to standardize gene sets from heterogeneous data sources. However, the integration of multiple heterogeneous terms remains incomplete, leading to high content-similarity [63] or even redundancy [64] in the gene set enrichment analysis and network analysis in practice [65–67].

To address the heterogeneities and inconsistencies of PAGs, we introduce super-PAG to represent clusters of PAGs and develop a new algorithm called Graph-Oriented Learning with Domain-Embedding Network (GOLDEN) fusion to generate super-PAG from regular PAGs. GOLDEN uniquely integrates graph embeddings and large language model (LLM)-based semantic embeddings, addressing redundancy and interpretability challenges, and features three advanced capabilities to mitigate biases inherent to individual embeddings. First, GOLDEN fusion introduces a new metric, the Connection Disparity Index (CDI), as a model-informed proxy for “clusterability.” Currently, the CDI is built on synthetic data from stochastic block models (SBMs) and degree-corrected stochastic block models (DC-SBMs) [68] to infer whether the network can be clustered into super-PAG based on the distribution of the clustering coefficient [69]. Second, GOLDEN fusion achieves optimal performance in super-PAG mining by integrating connection-based features from PAG–PAG networks and semantic-based features from PAG descriptions using a fusion model. Third, GOLDEN fusion provides summaries of the super-PAG by synthesizing descriptions from super-PAG members using LLMs. We tested GOLDEN fusion on the Gene Ontology (GO) network and demonstrated that GOLDEN fusion outperforms baseline models that utilize any individual information in generating super-PAG. We anticipate that super-PAG will play a pivotal role in biology and biomedicine by collecting, standardizing, and organizing gene set knowledge to facilitate multiscale biomedical data integration and analysis.

## Methods

### Overview design of GOLDEN fusion workflow

**Figure 1:**
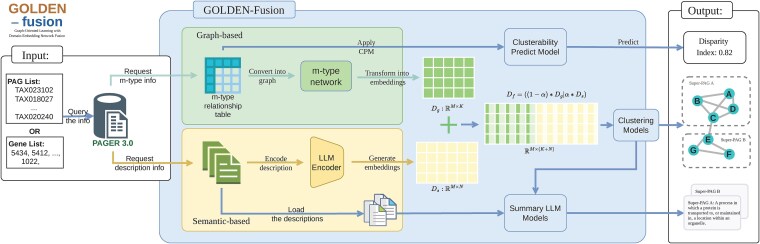
The framework of GOLDEN fusion. Input: The input can be either a PAG list identified by PAG IDs or a gene list identified by gene Entrez IDs. The system queries the PAGER database for m-type PAG–PAG relationship info and PAG description info. Graph-based embeddings: Converts a graph (m-type PAG–PAG relationship) into PAG embeddings, applies clustering methods to generate super-PAG, and uses a clusterability prediction model to evaluate whether the graph should be subdivided into meaningful subgraphs. Density-based embeddings: Encodes descriptions with an LLM encoder to create embeddings, which are then clustered. Output: Produces a CDI score, super-PAG, and summary descriptions for each super-PAG.

GOLDEN fusion is developed to enhance functional genomics analysis by creating abstract biological concepts that integrate both bio-semantic and network information. It employs a complex workflow that leverages graph transformations and machine learning models (Fig. 1).

The input for GOLDEN fusion can be either PAGs or a list of genes. PAGER 3.0 will retrieve co-membership-based (m-type) PAG–PAG relationships [70] as connection information and PAG descriptions as domain knowledge. The PAGER database has been instrumental in constructing PAG–PAG relationships and standardizing pathway gene sets for network biology [70]. If a PAG list identified by PAG IDs is provided, PAGER 3.0 will directly retrieve the m-type PAG–PAG relationships and PAG descriptions. If genes are provided as input, PAGER 3.0 will perform gene set enrichment analysis to generate significantly enriched PAGs, along with their m-type PAG–PAG relationships and PAG descriptions.

Subsequently, GOLDEN fusion performs a fusion procedure along two parallel paths: network-based information processing and domain knowledge-based information processing. The details of these processes are described in the following sections. The outputs of these two processes are two embedding matrices, *D_g_* and *D_s_*, representing the connection information and bio-semantic information, respectively. We performed early fusion of this information by concatenating their embeddings and applying a hyperparameter *α* to adjust the weight of each data source, resulting in a new embedding matrix *D_f_*. Clustering methods are then applied to *D_f_* to obtain the final clustering results, which form the putative super-PAG. These clustering results are also used as input for summarization models to generate descriptions for each cluster as an additional output.

To improve the robustness and accuracy of GOLDEN fusion’s clustering, we integrated a consensus clustering algorithm into its workflow. Consensus clustering is well-suited for aggregating the results of multiple clustering runs to provide a stable assessment of the clustering solution, mitigating sensitivity to initial conditions. Applied to the embedding fusion matrix *D_f_*, which combines connection-based and semantic-based features, consensus clustering helps determine the optimal number of clusters.

GOLDEN fusion generates three types of outputs: the CDI, serving as a proxy for clusterability; the putative super-PAG; and a summary description for each super-PAG.

### Generate synthetic networks for evaluating graph-based clusterability modeling

We generate synthetic networks using SBMs with various settings for the probabilities of inter-cluster and intra-cluster connections to evaluate the effectiveness of network-based clustering methods. Initially, we set the probability of inter-cluster connections at 0.5 and intra-cluster connections at 0.1 to test the performance of three main clustering algorithms: the Girvan–Newman Algorithm, the Louvain Algorithm, and Spectral Clustering.

Furthermore, to determine whether a PAG–PAG network is inherently amenable to clustering, we trained a predictive model on synthetic graphs and utilized its calibrated score as a CDI. Specifically, we generated two-block SBMs by exhaustively sweeping the intra- and inter-cluster connection probabilities from 0.1 to 1.0 in increments of 0.1. For each synthetic graph, we computed the clustering coefficient distribution (e.g. mean and variance) and obtained a supervision signal by clustering the graph and scoring the recovery of the planted partition using the Adjusted Rand Index (ARI). The resulting dataset (graph features with ARI-derived labels) was used to fit and compare four classifiers—logistic regression, support vector machine, k-nearest neighbors, and random forest—selecting the best model by cross-validated accuracy, precision, recall, and F1. The selected model’s calibrated output is reported as the CDI and is used to screen graphs and guide preprocessing (e.g. edge-weight thresholding) before applying GOLDEN fusion to real data. Additionally, to enhance the adaptability of the clusterability measurement, we generated synthetic networks using DC-SBMs [68] and trained with extended feature sets (described in the Supplementary Materials) using machine learning algorithms, including logistic regression, random forest, XGBoost, and multilayer perceptron (MLP), to derive CDI-β. The best-performing model was selected to estimate the probability of clusterability, reported as CDI-β.

### Generate benchmarking dataset using Gene Ontology Annotation network in PAGER 3.0

We evaluate GOLDEN fusion on the GOA m-type PAG–PAG network (biological process branch), using GOA *is-a* relations as ground truth. Inputs to GOLDEN fusion are either a list of PAG IDs or a gene list. For PAG inputs, PAGER 3.0 returns co-membership (m-type) PAG–PAG relationships and curated PAG descriptions. For gene inputs, PAGER 3.0 first performs gene set enrichment to obtain significantly associated PAGs, then returns their m-type links and descriptions. To construct fair, nontrivial test cases from the GOA hierarchy, we enumerate candidate groups subject to two constraints: (i) level-matched (nodes lie at the same shortest-path distance from a specified root), and (ii) parent-unique (no two nodes share the same immediate parent). We retain all groups meeting these criteria to form the benchmarks. Two size regimes were evaluated. (i) SPAGs 10–100, defined as GOA terms with larger than or equal to 10 but less than 100 child terms connected through hierarchical *is-a* relationships, representing candidate super-PAGs. This category included 127 two-member groups (pairs). (ii) SPAGs 5–10, defined as GOA terms with larger than or equal to 5 but less than 10 child terms, serving as auxiliary super-PAGs, with 300 two-member groups identified. For each group, GOLDEN fusion integrates the m-type edges and term descriptions of the child GOA terms within the two-member pair to explore GOA clusters. Model performance is evaluated by its ability to reconstruct putative super-PAGs that approximate the original parent GOA term (SPAG).

To benchmark GOLDEN fusion against the established similarity-based redundancy reduction and clustering methods, including semantic similarity, e.g. REVIGO [71] (default similarity cutoff is 0.7) and the Lin measure via GOSemSim [72, 73] (default similarity cutoff is 0.7), Jaccard-based redundancy control, e.g. ReCiPa [63] (max overlapping is 0.7, and low overlapping is 0.5), and overlap-based clustering, e.g. clusterProfiler [74] (default cutoff is 0.7), we applied all methods to the same GOA-derived benchmark datasets (SPAGs 5–10 and SPAGs 10–100) and evaluated using ARI and Normalized Mutual Information (NMI) against ground-truth labels, following the same repeated random-sampling protocol.

Additionally, to evaluate GOLDEN Fusion performance in pathway datasets, we construct the pathway groups based on the hierarchy of KEGG (*Homo sapiens*) and Reactome (*H. sapiens*). For KEGG, pathway descriptions and gene memberships were fetched via the KEGG REST API, and ground-truth labels were derived from the BRITE hierarchy at the B-level (subcategories such as “Carbohydrate metabolism” or “Nervous system”), yielding fine-grained but tractable categories. For Reactome, gene–pathway mappings were downloaded from Ensembl2Reactome_All_Levels.txt and hierarchy structure from ReactomePathwaysRelation.txt; ground-truth labels were obtained by walking each pathway up the parent–child hierarchy to its level-1 ancestor (direct child of a root), producing approximately 26 broad human categories (e.g. signal transduction, metabolism).

For each database, we constructed a pathway–pathway graph using the m-type relationship, computed Node2Vec graph embeddings (384-dimensional) and SentenceTransformer semantic embeddings (all-MiniLM-L6-v2, 384-dimensional), and combined them via the GOLDEN Fusion alpha-blend (α = 0.9 for KEGG, α = 0.9 for Reactome). Isolated nodes (zero graph degree) received zero-vector graph embeddings, ensuring full coverage without discarding any pathway. To obtain statistically robust estimates, we adopted a repeated random-sampling design: in each of 300 independent trials, 2 categories were drawn uniformly at random from all available categories, and up to 20 pathways were sampled without replacement from each selected category. Agglomerative Clustering with *k* = 2 set to the true number of drawn categories was then applied to the fused embeddings, and ARI and NMI were computed against the ground-truth labels. Results are reported as mean ± standard deviation over 300 trials, reducing variance from individual samples. Per-trial results are retained to ensure full reproducibility.

### Graph-based embedding generation

To identify higher-order structure among PAGs, we first build a graph based on shared gene membership (the m-type network). Each node represents a PAG, and edges between PAGs are weighted by the significance of their gene overlap, computed using a hypergeometric test. We denote this network as $\mathrm{G}=\left(\mathrm{V},\mathrm{E},\mathrm{W}\right)$ where *V* is the set of PAG nodes and *W​_ij_* is the weight between PAG *i* and PAG *j*, reflecting the statistical strength of their shared gene membership. We then learn a low-dimensional vector representation for each PAG using Node2Vec, which performs biased second-order random walks on the graph and trains a skip-gram model with negative sampling to produce embeddings. These graph-derived embeddings capture both direct and indirect connectivity patterns among PAGs and are later used as input for clustering.

We apply three clustering algorithms to these graph embeddings: *k*-means, agglomerative hierarchical clustering, and hierarchical density-based spatial clustering of applications with noise (HDBSCAN). Each offers a complementary view of the underlying structure.

- *k*-means assigns each PAG to one of *k* clusters by minimizing within-cluster variance; it is efficient and scalable.
- Agglomerative Clustering starts with each PAG as its own cluster and iteratively merges clusters based on similarity, producing a dendrogram; it does not require fixing *k* in advance and can reveal hierarchical organization.
- HDBSCAN identifies dense regions in the embedding space, can infer the number of clusters automatically, and can leave out poorly fitting PAGs as noise rather than forcing them into a cluster.

Using multiple clustering back-ends provides robustness and reduces bias toward any single geometric assumption (spherical clusters for *k*-means, linkage structure for agglomerative, local density structure for HDBSCAN). We assess clustering agreement with two complementary indices. The Adjusted Rand Index (ARI) measures overlap between the predicted and ground-truth partitions while correcting for chance; it ranges from −1 to 1 (random ≈ 0; perfect agreement = 1) and is sensitive to exact pairwise co-assignment. The NMI is an information-theoretic measure of shared information between partitions; it is bounded in [0,1] (no shared information = 0; identical partitions = 1) and is less affected by differences in the number or sizes of clusters. Together, ARI emphasizes pairwise label consistency, whereas NMI emphasizes global partition similarity, providing a more balanced evaluation.

### Semantic embedding generation

In parallel with the graph-based view, we model functional similarity between PAGs using their textual descriptions. Each PAG’s biological description is encoded into a dense vector using OpenAI’s text-embedding-3-small model. This produces a high-dimensional semantic embedding in which PAGs with similar biological functions are located near one another in the embedding space.

Because the raw description embeddings are higher dimensional than the graph embeddings, we align them for fusion. Specifically, we apply principal component analysis (PCA) to project the description embeddings down to the same dimension used for the Node2Vec graph embeddings, while retaining ~95% of the original variance. We then L2-normalize each projected vector. The resulting matrix, which we refer to as the semantic embedding matrix *D_s_*, reflects functional (semantic) similarity among PAGs and is directly comparable to the graph-based embedding matrix *D_g_*​.

We again apply *k*-means, Agglomerative Clustering, and HDBSCAN to these semantic embeddings to identify candidate super-PAGs based on functional descriptions alone. PAGs that cluster together in this space tend to share coherent biological themes in their annotations.

### Fusion and consensus clustering

Finally, we combine the graph-based and description-based embeddings into a unified representation to capture both topological and semantic relatedness. We form an embedding fusion for each PAG by taking a weighted linear blend of its Node2Vec vector and its description vector. Let *D*_g_ be the Node2Vec embedding matrix and *D*_s_ the PCA-aligned description embedding matrix. For a mixing weight α∈ [0,1], we define the embedding fusion matrix as follows:


$$ {D}_f=\left(1-\alpha \right){D}_g+\alpha{D}_s,\kern1em \alpha \in \left[0,1\right] $$

In practice, this means each PAG’s final feature vector is ${D}_{f,i}=\left(1-\alpha \right){D}_{g,i}+\alpha{D}_{g,i}$, a convex combination of the graph-derived features and description-derived features for PAG *i*. We perform clustering on these vectors (by default using *k*-means) to produce the final super-PAG groupings. The mixing coefficient α controls the influence of semantic versus network information: α = 0 corresponds to using only the graph embeddings, α = 1 uses only description embeddings, and intermediate values blend the two. We tune α by grid search on a validation split of the data, aiming to maximize clustering performance (e.g. mean ARI and cluster stability). The optimal α, along with the search range tested, is documented in our repository.

For the embedding fusion clustering, we also determine the number of clusters *k* in a data-driven way. Rather than fixing *k* a priori, we employ consensus clustering with the cumulative distribution function (CDF)/ΔCDF method to identify a stable number of clusters. In brief, we run the chosen clustering algorithm across multiple resampled subsets and evaluate the CDF of consensus indices; the point at which an increasing *k* yields diminishing returns (a plateau in the CDF) is selected as the appropriate cluster count. If multiple k-values are similarly stable, we favor the smaller *k* that achieves high consensus. This approach provides an unbiased criterion for choosing *k* and ensures that the resulting super-PAG are reproducible and well-separated. While *k*-means on the embedding fusion is our primary method for defining super-PAG, we also explored alternative clustering techniques as comparisons (summarized in Table 1). These include Spectral Clustering, Louvain community detection (on the graph), agglomerative clustering, HDBSCAN, and the Girvan–Newman algorithm for community finding. By comparing these alternatives, we verify that our fusion is robust and that the identified super-PAG structures are not an artifact of a single clustering method. Overall, this integrative pipeline—combining graph-oriented connection features and domain knowledge semantics features, and leveraging multiple clustering algorithms—allows us to discover coherent groups of gene sets in super-PAG that are supported by both gene overlap and functional description similarity, facilitating downstream biological interpretation of the clusters.

**Table 1:** Comparison of clustering methods.

**Method**	**Parameters**	**Scalability**	**Use case**	**Geometry**
*K*-Means	Number of clusters	Very large *n*, medium clusters	General-purpose	Euclidean distances
Spectral Clustering	Number of clusters	Medium *n*, small clusters	Few clusters	Graph distance
Agglomerative Clustering	Clusters/threshold, linkage, distance	Large *n* and clusters	Many clusters, non-Euclidean	Any pairwise distance
HDBSCAN	Minimum membership, point neighbors	Large *n*, medium clusters	Uneven sizes, outlier removal	Nearest points distances
Louvain	Resolution parameter	Very large *n*	Community detection (CD) in large networks	Graph-based modularity
Girvan–Newman	None (hierarchical edge betweenness)	Medium *n*	CD in small to medium networks	Graph-based modularity

### Utilizing large language models for PAG summarization

In our study, we harnessed the power of LLMs to facilitate the interpretation of PAGs by summarizing their descriptions. Given the often extensive and detailed functional descriptions associated with each PAG, manually synthesizing these into concise summaries for higher-level clusters, or super-PAG, can be laborious and inefficient. To address this, we employed LLMs to automate the summarization process, ensuring that each super-PAG is represented by a clear, informative sentence. We first concatenated all functional descriptions associated with each PAG within a cluster into a single, comprehensive text block, ensuring that LLM receives full contextual information about the cluster’s biological functions. Using this concatenated text, the LLM generated a summary by processing the input and distilling it into a coherent sentence that encapsulates the essence of the cluster’s biological functions. This summary provides a high-level overview of the super-PAG, facilitating easier interpretation by researchers. Given the inherent input size limitations of LLMs, we optimized our method by monitoring the length of the concatenated descriptions. When the input approached the model’s capacity, we applied additional summarization steps to further condense the text before feeding it into the LLM. This process ensured that all relevant information was retained while staying within the model’s input limits.

### CDI-based separation of SPAG-to-SPAG pairs validated by functional independence

To evaluate SPAG-to-SPAG pairs exhibiting both high CDI and ARI scores, we assessed functional relevance by examining the GOA term names and descriptions, with supporting evidence. These assessments serve to contextualize CDI-based separation results by examining whether SPAG-to-SPAG pairs correspond to biologically independent or overlapping processes. Each pair was categorized as Strong, Moderate, or Weak based on biological proximity derived from shared pathways, hierarchical relationships, and conceptual linkage within cellular or organismal processes. The assigned relevance level reflects how closely the paired GOA terms contribute to the same biological mechanism, pathway, or regulatory context. Pairs classified as Strong demonstrate clear mechanistic relationships, including (i) involvement in the same biological pathway or process, (ii) representation of sequential or hierarchical steps within the same mechanism (e.g. activation → differentiation → effector function), or (iii) evidence of direct regulatory or causal dependency. Pairs classified as Moderate exhibit related but not strictly dependent biological functions, including cases where terms (i) operate in related processes without obligate interaction; (ii) share overlapping roles, pathways, or cell types; (iii) function within the same biological system (e.g. immune, metabolic, reproductive) at different regulatory levels; or (iv) interact functionally but do not represent consecutive steps in the same pathway. Pairs classified as Weak show limited or indirect functional relationship, such as when terms (i) belong to distinct biological systems or domains; (ii) lack shared pathways, gene annotations, or biological rationale for linkage; (iii) occur in different compartments, developmental stages, or tissue contexts; or (iv) share only broad conceptual similarity (e.g. both regulatory terms) without a meaningful mechanistic connection.

**Figure 2:**
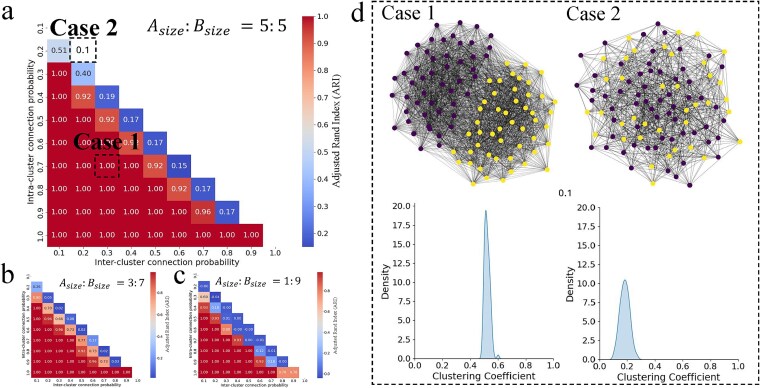
Heatmap of ARI comparing clusterability across three settings. The node composition of two subgraphs is presented with proportions of (a) 0.5 and 0.5, (b) 0.7 and 0.3, and (c) 0.9 and 0.1 generated by an SBM. Higher ARI values indicate more accurate recovery of ground-truth clusters. (d) The two cases illustrate the distribution of clustering coefficients, comparing a clusterable scenario (Case 1) with an unclusterable scenario (Case 2) using Spectral Clustering. An ARI of 0.7 is used as the cutoff for determining clusterability to train the classification models.

## Results

### Clustering back-ends and experimental setup

For each representation—graph-only (Node2Vec), description-only (OpenAI text embeddings), and the fusion (concatenation)—we clustered the resulting feature matrix with three back-ends: Agglomerative Clustering, *k*-means, and HDBSCAN. Agglomerative Clustering (scikit-learn default settings) was run with a fixed number of clusters *k*, identical to the *k* used for *k*-means. *k*-means used *k = num_clusters* with a fixed initialization seed. HDBSCAN was run in a parameterization suited to discovering uneven, potentially sparse structure (min_cluster_size = 5, gen_min_span_tree = True), allowing the number of clusters to be determined automatically; points deemed insufficiently dense were labeled as noise (−1). Unless stated otherwise, the same settings were applied across benchmarks (SPAGs 5–10 and SPAGs 10–100) and information sources, and performance was evaluated with ARI and NMI.

### Exploring clusterability: the performance of clusterability predictive model

In SBM networks with different probability settings: (inter-cluster versus intra-cluster) as (0.3, 0.1), (0.5, 0.01), (0.6, 0.4), and (0.8, 0.1) across varying configurations (see Fig. S1), we found that Spectral Clustering consistently outperformed the other two algorithms in accurately detecting the underlying cluster structures. We selected Spectral Clustering as the primary method for analyzing synthetic datasets in the subsequent sections of our study. The clusterability predictive model was designed to classify synthetic networks into unclusterable and clusterable categories with an accuracy of 0.88 and an F1 score of 0.9.

The ARI was utilized as the standard for gauging clusterability in the synthetic networks. We employed Spectral Clustering, the best-performing algorithm reported in the connection-based clustering, to predict clusters and evaluate their agreement with the ground truth clusters. As shown in the ARI measurement heatmap in Fig. 2, a clear boundary emerges that separates networks that can be reliably clustered from those that cannot. Based on this observation, we set an ARI threshold of 0.7 (Fig. 2a–c) as a conservative criterion for defining reliable cluster recovery. We further verified that consistent CDI–functional relevance trends were maintained across nearby thresholds (ARI ≈ 0.65–0.75), demonstrating robustness to moderate cutoff variation. This thresholding step transforms network clusterability, as assessed by Spectral Clustering, into a binary classification problem.

We developed a clusterability predictive model described in Algorithm 1. The input of the model is the mean and variance of clustering coefficient distribution and the output is the CDI. In Fig. 2d, we can intuitively identify the differences in clustering coefficient distribution between unclusterable and clusterable networks. The unclusterable network displayed a normal distribution with low clustering coefficients (0.2). In contrast, the clusterable network exhibited a high clustering coefficient (0.5 to 0.6) with a bimodal distribution. Specifically, we employed logistic regression, support vector machines (SVM), random forest, and k-nearest neighbors (KNNs) models. The random forest model demonstrated superior performance with an F1-score of 0.90, signifying a high balance between precision and recall (Table 2). Logistic regression also showed commendable results, particularly in terms of recall.

**Table 2:** Comparison of different methods based on performance metrics. KNN represents the k-nearest neighborhood model.

**Methods**	**Accuracy**	**Precision**	**Recall**	**F1**
Logistic regression	0.76	0.73	**1**	0.84
SVM	0.67	0.66	**1**	0.79
Random forest	**0.88**	**1**	0.81	**0.9**
KNN	0.76	**1**	0.63	0.77

Bold values indicate the best performance among all compared methods.

To test whether CDI is useful beyond synthetic data, we applied the trained random forest model to the same GOA-derived benchmarking datasets used to evaluate GOLDEN fusion. We observed that CDI correlated with downstream clustering performance on real biological groups. In cases where CDI was high (for example, CDI ≈ 0.93 for a PAG pair whose members map to clearly distinct biological concepts), GOLDEN fusion produced two well-separated clusters with high agreement to the GOA *is-a* ground truth (ARI ≈ 1.0). In contrast, when CDI was low (for example, CDI ≈ 0.40 for a PAG pair whose members share very similar biological annotations), GOLDEN fusion struggled to cleanly split the pair, and agreement with the ground truth dropped (ARI ≈ 0.44).

In the CDI-β performance test, we found that XGBoost was the best-performing model across all cluster-size settings (Table S1). Compared to logistic regression and neural network (MLP), XGBoost exhibited greater robustness to increasing cluster complexity, maintaining stable performance as cluster size increased. While random forest showed competitive performance in small clusters, its performance was less consistent across larger cluster settings. Overall, these results indicate that XGBoost provides the most reliable and scalable estimation of clusterability to derive CDI-β.

Overall, the clusterability predictive model provides a quantitative method for evaluating the validity of clustering algorithms in network clusterability problems. This approach is especially valuable for assessing complex network structures where discerning clear clusterability boundaries is critical.

**Algorithm 1** An algorithm to develop a prediction model to determine the clusterability of a graph.

Given:

- A set of feature vectors of mean and std $\boldsymbol{X}=\left|{\boldsymbol{x}}_1,{\boldsymbol{x}}_2,\dots, {\boldsymbol{x}}_n\right|$, where ${\boldsymbol{x}}_i\in{R}^d$.
- A set of ARI score variables $\boldsymbol{y}=\left|{y}_1,{y}_2,\dots, {y}_n\right|$, where ${y}_i\in \left[0,1\right]$.
- A threshold value $\tau$ for binarization (commonly $\tau =0.7$).

Binarize target variables:


$$ {y}_i^{\prime }=\left\{\begin{array}{c}1\kern0.75em if\ {y}_i\ge \tau \\{}0\kern0.5em otherwise\end{array}\right.,{\forall}_{\mathrm{i}}\in \left|1,2,\dots, n\right| $$

Partition data: Split $\boldsymbol{X}$ and ${\boldsymbol{y}}^{\prime }$ into training and test sets, $\left({\boldsymbol{X}}_{train},{\boldsymbol{y}}_{train}^{\prime}\right)$ and $\left({\boldsymbol{X}}_{test},{\boldsymbol{y}}_{test}^{\prime}\right)$, using a 75% and 25% split.

Model training: Train a binary classification model $f$ using $\left({\boldsymbol{X}}_{train},{\boldsymbol{y}}_{train}^{\prime}\right)$.

Prediction: Use the trained model $f$ to predict labels for ${\boldsymbol{X}}_{test}$, obtaining predicted labels ${\hat{\boldsymbol{y}}}_{test}$.

### Graph-based clustering performance

Using only the graph signal (Node2Vec embeddings of the m-type PAG–PAG network), we compared *k*-means, Agglomerative Clustering, and HDBSCAN on both benchmarks. Unless otherwise stated, we used typical Node2Vec hyperparameters for graph embeddings: walk length = 80, walks per node = 10, context window = 10, negative samples = 5, return parameter *p* = 1.0, and in–out parameter *q* = 0.25. We examined two embedding dimensions (*d* = 64 and *d* = 256), and the resulting node embedding matrix was L2-normalized row-wise.

**Figure 3:**
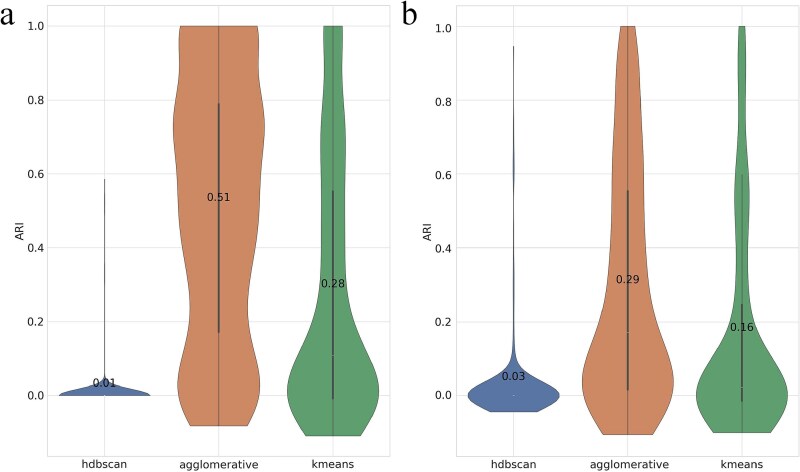
Recovery performance of parent GOA terms measured by ARI, comparing three clustering algorithms—HDBSCAN, agglomerative, and *k*-means—using graph-based embeddings on (a) SPAGs 5–10 and (b) SPAGs 10–100. Numeric labels indicate mean values.

- SPAGs 5–10 (Fig. 3a). Agglomerative Clustering achieved the best recovery of the *is-a* structure with a mean ARI of 0.51. *k*-means was second (0.28), providing stable but less accurate partitions. HDBSCAN clustered a few items confidently and remained near zero (0.01), indicating density thresholds in the Node2Vec space were too strict for these small graphs.
- SPAGs 10–100 (Fig. 3b). Absolute scores decreased on the larger, more heterogeneous graphs, but Agglomerative Clustering remained strongest (0.29). *k*-means again ranked second (0.16). HDBSCAN showed a long-tailed distribution with rare high scores but a low mean (0.03), reflecting unreliable behavior under uneven densities.
  Across both benchmarks, Agglomerative Clustering is the most reliable back-end for the graph-only baseline; *k*-means is a reasonable and stable alternative; HDBSCAN is generally unsuitable for these Node2Vec representations.

### Semantic-based clustering performance

**Figure 4:**
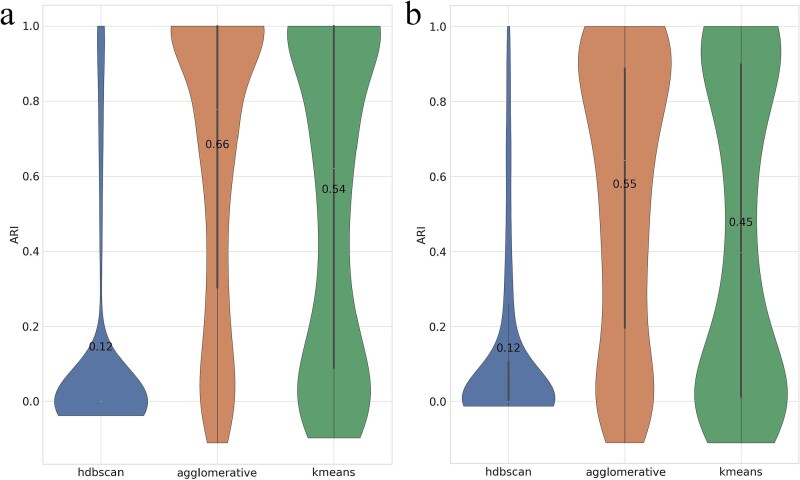
Recovery performance of parent GOA terms measured by ARI, comparing three clustering algorithms—HDBSCAN, agglomerative, and *k*-means—using semantic-based embeddings on (a) SPAGs 5–10 and (b) SPAGs 10–100. Numeric labels indicate mean values.

Using only description embeddings from OpenAI’s text-embedding-3-small, we evaluated *k*-means, Agglomerative Clustering, and HDBSCAN on the same benchmarks (Fig. 4).

- SPAGs 5–10 (Fig. 4a). Agglomerative Clustering delivered the highest mean ARI (0.66), indicating strong alignment between semantic similarity and the *is-a* structure at small node counts. *k*-means also performed well (0.54), while HDBSCAN trailed with a low mean (0.12), suggesting insufficient density separation in the text-embedding space.
- SPAGs 10–100 (Fig. 4b). The performance decreased modestly with larger clusters, yet the ordering persisted: Agglomerative Clustering remained best (0.55), followed by *k*-means (0.45), with HDBSCAN again lowest (0.12).

Across both datasets, Agglomerative Clustering is the most reliable description-only back-end; *k*-means provides a competitive and stable baseline; HDBSCAN underperforms due to weak density contrasts in the high-dimensional text-embedding space.

### GOLDEN fusion outperforms single-source baseline

To contextualize the fusion model (graph + description), we compared two single-source baselines on the GOA benchmarks: a graph-only baseline that clusters PAGs using the m-type PAG–PAG network, and a semantic-only baseline that clusters PAGs using OpenAI text embeddings of PAG descriptions. Each baseline was evaluated with *k*-means, Agglomerative Clustering, and HDBSCAN, and scored by ARI against the GOA *is-a* ground truth. Across both SPAGs 5–10 and SPAGs 10–100, GOLDEN fusion (the weighted fusion) achieved higher ARIs than either baseline, indicating that combining network topology and description semantics yields more faithful recovery of the ontology structure.

**Figure 5:**
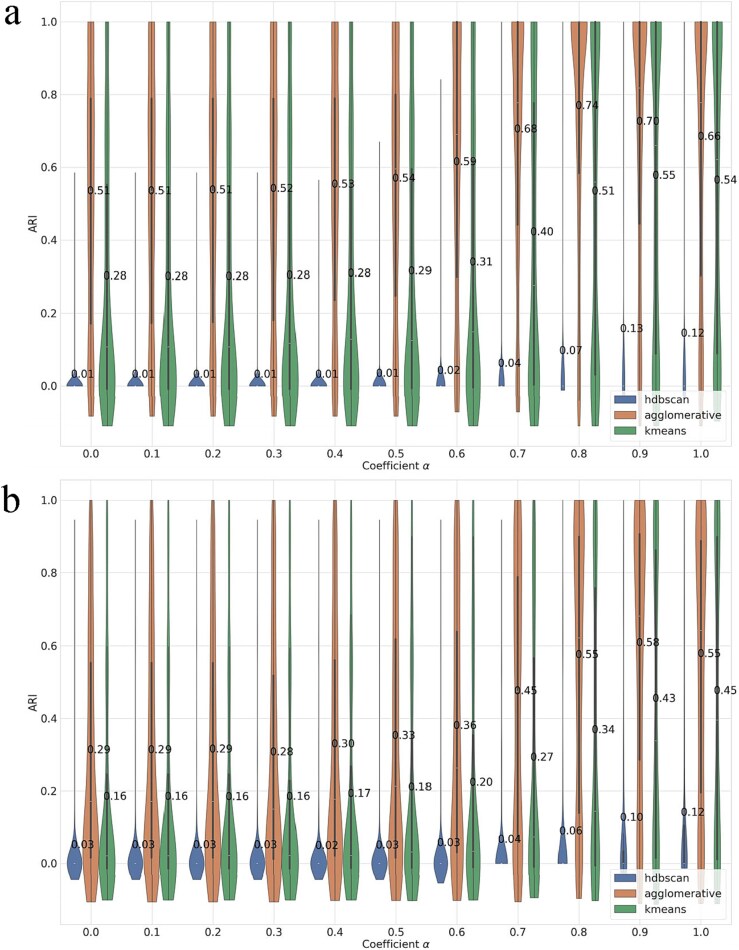
Coefficient optimization of parent GOA term recovery, measured by ARI, comparing three clustering algorithms—HDBSCAN, agglomerative, and *k*-means—using embedding fusion on (a) SPAGs 5–10 and (b) SPAGs 10–100. Numeric labels indicate mean values.

To determine the fusion weight α and a cost-effective representation size, we (i) swept α ∈ [0,1] and (ii) varied dimensions by training two Node2Vec models (64-d and 256-d) and pairing each with OpenAI embeddings at 64, 256, 512, and 1024 (projected via PCA to match the graph dimension prior to fusion). The best configurations consistently favored the semantic channel (peak performance at α ≈ 0.8, i.e. ~20% graph + ~80% semantic). Across both SPAGs 5–10 and 10–100, the 256–512 pairing (256-d Node2Vec + 512-d OpenAI) delivered the highest mean ARI overall with strong stability. We observed a slight ARI gain with the 256–1024 pairing on the larger SPAGs 10–100 benchmark, but this came at a noticeably higher computational cost. Considering the accuracy–efficiency trade-off, we recommend 256–512 as the default dimensional setting, reserving 256–1024 for scenarios where marginal improvements justify additional compute (see Fig. 5 and Table 3). In comparison with established similarity-based redundancy reduction and clustering methods, GOLDEN fusion consistently and substantially outperformed REVIGO, GOSemSim, ReCiPa, and clusterProfiler across both SPAGs 5–10 and SPAGs 10–100 datasets (Table S2). Particularly, for the graph-based evaluation along, m-type network embeddings yield ARI = 0.51 (SPAGs 5–10) and ARI = 0.29 (SPAGs 10–100) (Fig. 3), outperforming ReCiPa (ARI = 0.07 and 0.01) and clusterProfiler (ARI = 0.43 and 0.24), respectively. For the KEGG and Reactome pathway datasets, GOLDEN fusion demonstrates a little higher performance on Reactome compared to KEGG (Table S3). This difference is consistent with Reactome’s finer-grained hierarchy and richer semantic annotations, which better support joint graph–semantic integration.

**Table 3:** Optimization of PAG recovery performance using different combinations of connection embedding and semantic embedding dimensions. Optimal performance was achieved using a 256-dimensional connection embedding and a 512-dimensional semantic embedding.

**Super-PAG dataset**		**SPAGs 5–10**								**SPAGs 10–100**							
Connection embedding		64				**256**				64				**256**			
Semantic embedding		1024	512	256	64	1024	**512**	256	64	1024	512	256	64	1024	**512**	256	64
agglomerative	Alpha	0.8	0.8	0.8	0.8	0.8	0.8	0.8	0.8	0.9	0.9	0.9	0.8	0.9	0.9	0.9	0.8
	ARI_Mean	0.72	0.73	0.73	0.72	**0.75**	0.74	0.74	0.74	**0.58**	**0.58**	**0.58**	**0.58**	**0.58**	**0.58**	**0.58**	**0.58**
	NMI_Mean	0.73	0.74	0.73	0.72	**0.76**	0.75	0.75	0.74	**0.56**	0.55	0.55	0.50	**0.56**	**0.56**	**0.56**	0.50
kmeans	Alpha	0.9	0.9	1	1	0.9	0.9	0.9	0.9	1	1	1	1	1	1	1	1
	ARI_Mean	0.12	0.12	0.12	0.11	**0.13**	**0.13**	**0.13**	0.12	0.11	**0.12**	**0.12**	0.11	0.11	**0.12**	**0.12**	0.11
	NMI_Mean	0.13	0.13	0.12	0.07	**0.14**	**0.14**	0.13	0.07	0.14	**0.15**	0.14	0.11	0.14	**0.15**	0.14	0.11
hdbscan	Alpha	0.9	0.9	0.9	0.9	0.9	0.9	0.9	0.9	1	1	0.9	0.9	1	1	1	0.9
	ARI_Mean	0.54	**0.55**	0.53	0.49	**0.55**	**0.55**	0.53	0.48	**0.45**	**0.45**	0.44	0.41	**0.45**	**0.45**	0.44	0.41
	NMI_Mean	0.58	**0.59**	0.57	0.53	0.58	**0.59**	0.56	0.51	**0.46**	**0.46**	0.45	0.42	**0.46**	**0.46**	0.44	0.42

Bold values represent the best performance under specific parameter settings across the compared methods.

### The PAG summarization captures important information in the benchmarking dataset

In this study, we evaluated 10 models for summarizing PAG descriptions, including GPT-4.1, GPT-3.5, BART-based variants, SciFive [75], Pegasus [76], BioGPT [77], BioBART [78], BioRAgent [79], and Flan-T5 [80]. GPT-4.1 and GPT-3.5 are instruction-tuned, decoder-only LLMs that perform well in zero-shot abstractive summarization; both were applied in a zero-shot, single-sentence summarization setting. Encoder–decoder transformer models, including BART variants, SciFive, and Pegasus, served as strong supervised baselines. Across both benchmark datasets, GPT-4.1 achieved the highest cosine similarity, indicating the strongest alignment with original PAG descriptions. Specifically, on SPAGs 5–10, GPT-4.1 reached a cosine similarity of 0.71 ± 0.10, followed by GPT-3.5 (0.71 ± 0.12) and BART-CNN (0.65 ± 0.17). On SPAGs 10–100, GPT-4.1 again led with 0.72 ± 0.13, followed by GPT-3.5 (0.69 ± 0.12) and SciFive (0.58 ± 0.15). In contrast, encoder–decoder models such as SciFive, BART-CNN, and BioBART achieved higher ROUGE-1 F and ROUGE-L F scores [81], reflecting stronger lexical overlap with reference descriptions, albeit with lower semantic alignment as measured by cosine similarity. Models such as Flan-T5 and BioGPT showed comparatively lower performance across both semantic and lexical metrics. Collectively, these results suggest that while supervised summarization models better preserve surface-level textual overlap, larger instruction-tuned LLMs (GPT-4.1 > GPT-3.5) provide superior semantic fidelity, making them more suitable for generating biologically meaningful summaries of PAGs (Table 4).

**Table 4:** Comparison of PAG description summarization performance across ten methods (GPT-4.1, GPT-3.5, BART-CNN, SciFive, Pegasus, BioGPT, BioBART, BART-XSum, Flan-T5, and BioRAGent) on SPAGs 5–10 and SPAGs 10–100 datasets.

**Evaluation Dataset**	**Model**	**Cosine similarity**	**ROUGE-1 F**	**ROUGE-L F**
SPAGs 5–10	GPT-4.1	**0.71 ± 0.10**	0.34 ± 0.09	0.24 ± 0.07
	GPT-3.5	0.71 ± 0.12	0.28 ± 0.11	0.22 ± 0.09
	BART-CNN	0.65 ± 0.17	0.43 ± 0.16	0.35 ± 0.16
	SciFive	0.63 ± 0.15	**0.45 ± 0.15**	0.36 ± 0.15
	Pegasus	0.58 ± 0.31	0.40 ± 0.30	**0.37 ± 0.30**
	BioGPT	0.57 ± 0.18	0.27 ± 0.14	0.22 ± 0.10
	BioBART	0.55 ± 0.09	0.42 ± 0.14	0.36 ± 0.14
	BART-XSum	0.52 ± 0.22	0.28 ± 0.13	0.21 ± 0.12
	Flan-T5	0.24 ± 0.08	0.17 ± 0.05	0.13 ± 0.05
	BioRAGent	0.55 ± 0.19	0.29 ± 0.10	0.20 ± 0.08
SPAGs 10–100	GPT-4.1	**0.72 ± 0.13**	0.35 ± 0.12	0.24 ± 0.09
	GPT-3.5	0.69 ± 0.12	0.28 ± 0.09	0.23 ± 0.08
	SciFive	0.58 ± 0.15	**0.42 ± 0.15**	0.31 ± 0.13
	BART-CNN	0.56 ± 0.16	0.39 ± 0.16	0.32 ± 0.17
	BART-XSum	0.54 ± 0.19	0.31 ± 0.14	0.23 ± 0.13
	Pegasus	0.53 ± 0.27	0.28 ± 0.22	0.24 ± 0.22
	BioBART	0.52 ± 0.08	0.40 ± 0.13	**0.32 ± 0.15**
	BioGPT	0.43 ± 0.17	0.24 ± 0.16	0.19 ± 0.13
	Flan-T5	0.28 ± 0.11	0.19 ± 0.08	0.14 ± 0.08
	BioRAGent	0.47 ± 0.20	0.27 ± 0.11	0.17 ± 0.07

Bold values indicate the best performance achieved among the compared models on the SPAGs 5–10 and SPAGs 10–100 datasets.

### High CDI scores correspond to functionally independent SPAG pairs

Across both SPAG-to-SPAG pair evaluations, a clear pattern emerges when comparing functional relevance distributions between CDI-high and CDI-low groups. SPAG pairs with high CDI values show a substantially greater proportion of weak functional relevance, whereas CDI-low groups are more enriched for strong or moderate functional relationships. In the example list of SPAG (5–10) to SPAG (5–10) pairs, weak functional relevance accounts for 40.0% of CDI-high pairs but only 9.1% of CDI-low pairs (Δ = −30.9%), indicating that high CDI values are more likely to separate biologically independent or weakly related GO processes (Table 5, Figs S4 and S5). Conversely, CDI-low pairs show a markedly higher proportion of moderate functional relevance (54.5% versus 30.0%, Δ = +24.5%), suggesting that lower CDI values are associated with closer biological function or pathway proximity. A similar trend is observed in the example list of SPAG (10–100) to SPAG (5–100) pairs. Weak relationships remain more frequent in CDI-high pairs (40.0%) compared with CDI-low (28.6%, Δ = −11.4%), while strong relationships are more represented in CDI-low pairs (42.9%) than CDI-high (30.0%, Δ = +12.9%).

**Table 5:** The example lists of SPAG to SPAG pairs with CDI high and CDI low, and their functional relevance. CDI-high SPAG–SPAG pairs are enriched for weak functional relevance, consistent with functional independence.

**A list of SPAG to SPAG pairs**	**Functional relevance**	**CDI high**	**CDI low**	**Difference**
SPAG (5–10) to SPAG (5–10)	Strong	30.0%	36.4%	6.4%
	Moderate	30.0%	54.5%	24.5%
	Weak	40.0%	9.1%	−30.9%
SPAG (10–100) to SPAG (10–100)	Strong	30.0%	42.9%	12.9%
	Moderate	30.0%	28.6%	−1.4%
	Weak	40.0%	28.6%	−11.4%

### Case study of chronic myeloid leukemia tyrosine kinase inhibitor resistant versus sensitive

**Figure 6:**
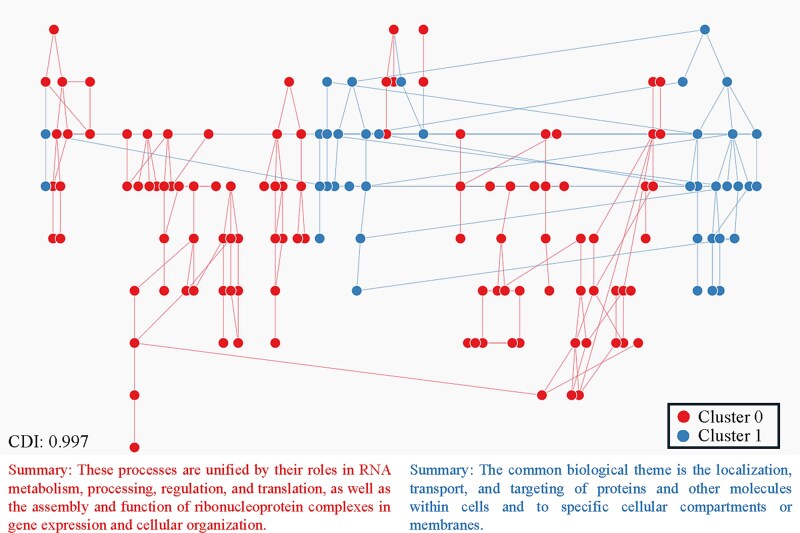
Visualization of the two super-PAG generated by GOLDEN fusion, with colored dots representing two distinct clusters. The GOs are enriched from a list of differentially expressed genes (DEGs) in a case study comparing CML TKI resistant versus sensitive samples.

GOLDEN fusion was applied to a case study as an illustrative example to demonstrate how GOA PAGs are derived from gene set enrichment analysis. The input consisted of a list of differentially expressed genes in chronic myeloid leukemia (CML) samples resistant and sensitive to tyrosine kinase inhibitors (TKIs) [82, 83]. GOLDEN fusion identified two super-PAG structures within the selected PAGs, and consensus clustering showed that the solution stabilized at *k* = 2 and CDI = 0.997, corresponding to the highest consensus index with a high probability to be clusterable (Fig. S2 and Fig. 6). One cluster summarized the concepts related to the development of resistance to chemotherapy drugs, rendering the treatment ineffective in inhibiting cancer cell proliferation or inducing cell death. This discovery has been repeatedly validated through experimental results from BCR-ABL kinase-independent pathways that promote CML cell survival and proliferation, bypassing TKI inhibition [84, 85]. Conversely, the other cluster consisted of DNA or RNA functions that continued to respond favorably to therapeutic agents (mRNA splicing, maturation of LSU-rRNA, DNA-damage lesion, etc.). Previous studies showed that aberrant RNA splicing in BCR-ABL variants (BCR-ABL35INS) has been associated with poor response to TKI treatment (Imatinib) [86–88]. Additionally, by retrieving the *is-a* relationships to construct the GOA tree, the two major GOA clusters were recovered in different branches of the tree (Fig. 6 and supplemental material—the interactive network). This real-world application of our framework highlights its potential utility in distinguishing differential cellular responses to treatment in leukemia.

## Discussion

We demonstrated GOLDEN fusion as a novel way of capturing and representing biologically and functionally important super-PAG from regular PAGs. Routine generation of multi-omics data has greatly increased the diversity of PAGs in the PAGER database. It is critical to perform GOLDEN fusion to ensure that super-PAG clearly and comprehensively represents the results of GNPA. We introduced a critical metric, the clustering coefficient distribution index, for gauging “Clusterability” in PAG networks and evaluated the super-PAG construction using the SBM. The clustering coefficient metric enables the evaluation of any type of network to be clustered or not and further provides guidance for further refining networks with a higher edge confidence cutoff. This choice ensures robust and reliable identification of community structures, especially in networks characterized by complex modularity and connectivity patterns.

As the complexity of the PAG content integration increases while considering the PAG–PAG network and PAG semantic description, GOLDEN fusion implements the embedding fusion from the m-type PAG–PAG relationship and the PAG description to balance the knowledge-based information and gene memberships to achieve the best performance. GOLDEN fusion will be flexible in integrating additional information layers, such as embeddings from gene-miRNA relationships, gene set functional inference similarity, etc. To enhance the interpretability of super-PAG, we performed description extraction and summarization of gene sets with state-of-the-art LLM techniques.

By combining multiple sources of information, GOLDEN fusion offers a powerful and flexible framework for functional genomics analysis, facilitating deeper insights and more accurate interpretations of gene set relationships. Particularly, the semantic-based clustering outcomes were analyzed to assess the effectiveness of the LLM-based feature representation. The results revealed that the embedding generated by the OpenAI’s text-embedding-3-small model enhanced clustering quality, as indicated by improved cluster coherence and separation. Thus, GOLDEN fusion provided deeper insights into the relationships between gene sets and offered a more nuanced understanding compared to traditional clustering methods based on simple numerical features. The integration of semantic features through LLMs, therefore, holds significant promise for advancing the interpretability and accuracy of bioinformatics analyses.

GOLDEN fusion can be extensively used for any gene set summarization based on multi-omics data and single-cell data. The representation of the gene set can be enriched by considering different types of activities from various omics sources, such as mutation burden [89], transcriptional activity [90], proteomics activity [91], and metabolic activity [92], facilitating deeper insights and more comprehensive interpretations of gene sets and putative gene set clusters. All gene set activities can be converted into embeddings to form different super-PAG for different levels of integration. Additionally, with the prevalence of single-cell data analysis, cell-type-specific and cluster-specific super-PAG can be generated using cell-based multi-omics profiles [93]. Therefore, GOLDEN fusion has the potential for broad data implementation and plays a pivotal role in examining non-redundant and comprehensively condensed information in GNPA, making it extremely useful for diverse applications in multi-omics and single-cell studies. Beyond CML, GOLDEN fusion would benefit from additional experimental or literature-based validation of identified super-PAG.

For best practices in GNPA with super-PAG, GOLDEN fusion is poised for potential improvements. In connection-based clustering, the m-type PAG–PAG relationships were used to generate connection-based embeddings. Using a higher threshold can result in better pattern representation but may lead to a tradeoff with a lower network coverage rate, leaving some PAGs uncovered in a PAG–PAG network. One solution could be locally adaptive smoothing with signal denoising [94]. Additionally, the complexity of network modeling increases when integrating multi-modality relationships, such as transcriptional regulatory relationships and post-transcriptional regulatory relationships. Co-embedding of PAG–PAG relationships, PAGs, and nodes can be implemented to model multi-dimensional edge features [95]. In the semantic-based clustering of embeddings from PAG descriptions using LLMs, the current model can be substituted with other LLMs, including the newest ChatGPT versions [96] and LLM2Vec [97]. LLM-generated summaries are intended to support interpretation and navigation of super-PAGs and do not replace curated ontology definitions or expert biological annotation. Therefore, the current implementation of GOLDEN fusion is limited by the absence of human curation for validating LLM-generated summaries. In future work, we plan to develop an interactive platform that incorporates expert feedback to refine and validate generated terms, enabling improved performance through human-in-the-loop curation and enhanced prompt engineering. Currently, super-PAG are regarded as flattened one-level structures above regular PAGs. For multi-omics integration, the connection-based and semantic-based embeddings can be enhanced through matrix addition when the dimensions are the same. The potential benefit of this approach is that setting the coefficient to 0 or 1 can yield exact results from the single embedding. In future work, GOLDEN fusion will be implemented to recover or construct a multi-level architecture using auto-weighted embedding fusion learning. Although GOA is used as the primary benchmarking resource in this study, the GOLDEN fusion framework is ontology-agnostic and requires only (i) gene membership information and (ii) textual descriptions. As such, it can be readily applied to other pathway databases, disease signatures, cell-state programs, and emerging multi-omics gene set collections, extending its utility beyond GO-based analyses.

## Conclusion

Overall, GOLDEN fusion takes advantage of network biology and LLM to generate super-PAG with enhanced interpretability and quality for GNPA. It shows potential in comprehensive data integration and knowledge abstractive summarization. We expect that super-PAG generated from GOLDEN fusion will become an extensive and publicly accessible source of nonredundant gene sets with curated biological context. Meanwhile, GOLDEN fusion provides a scalable framework with the potential to improve and enhance the biologists’ experience in GNPA.

**Key Points**

- GOLDEN fusion integrates graph-based connectivity and LLM-derived semantic embeddings to generate concise, nonredundant super-PAGs, improving interpretability in functional genomics and gene set network analysis.
- A novel Connection Disparity Index (CDI) is introduced to interpret a model-informed proxy of clusterability, enabling principled screening of PAG–PAG networks and improving the reliability of downstream clustering.
- On Gene Ontology benchmarks, GOLDEN fusion consistently outperforms graph-only and semantic-only baselines, achieving higher Adjusted Rand Index (ARI) and Normalized Mutual Information (NMI) in recovering hierarchical structure.
- GOLDEN fusion outperforms established similarity-based redundancy reduction and clustering methods, including semantic similarity, Jaccard-based redundancy control, and overlap-based clustering.
- LLM-based summarization provides exploratory descriptions for super-PAGs, further facilitating interpretation from translational and systems biology perspectives.

## Supplementary Material

Supplementary_material_bbag244

## Data Availability

The source codes and datasets are publicly accessible at https://github.com/ai-pharm-AU/GOLDEN/.
