# Supplementary material for: GOLDEN fusion: a graph-oriented learning with domain-embedding network fusion for generating super gene sets in functional genomics
Source: Brief Bioinform. 2026 May 25;27(3):bbag244. doi: 10.1093/bib/bbag244 (PMC13200546; doi:10.1093/bib/bbag244)
Supplement: Supplementary_material_bbag244 [file supplementary_material_bbag244.zip › GOLDEN_fusion_suppl_final_bbag244.docx]

Supplementary GOLDEN Fusion: A Graph-Oriented Learning with Domain-Embedding Network Fusion for Generating Super Gene Sets in Functional Genomics

Qi Li^1^, Cody Nichols^2^, Robert S Welner^3^, Jake Y. Chen^4^*^*^*, Wei-Shinn Ku^2^*^*^*, and Zongliang Yue^5^*^*^*

^1^Mathematics and Computer Science Department, School of Natural Sciences Mathematics & Business, Fisk University, TN, USA

^2^Computer Science and Software Engineering Department, Samuel Ginn College of Engineering, Auburn University, Auburn, AL, USA

^3^Hematology & Oncology, Heersink School of Medicine, University of Alabama at Birmingham, AL, USA

^4^Biomedical Informatics and Data Science, Heersink School of Medicine, University of Alabama at Birmingham, AL, USA
^5^Department of Health Outcomes Research and Policy, Harrison College of Pharmacy, Auburn University, AL, USA

^∗^Corresponding authors: Zongliang Yue [zzy0065@auburn.edu](mailto:zzy0065@auburn.edu), Wei-Shinn Ku [wzk0004@auburn.edu](mailto:wzk0004@auburn.edu), Jake Chen [jakechen@uab.edu](mailto:jakechen@uab.edu)

# Evaluation Methods

Mathematically, ARI is defined as follows:

$\mathrm{ARI}=\frac{\sum_{ij} (\begin{matrix} n_{ij} \\ 2 \end{matrix})-\left[ \sum_{i} \left( \begin{matrix} a_{i} \\ 2 \end{matrix} \right)\sum_{j} \left( \begin{matrix} b_{j} \\ 2 \end{matrix} \right) \right]/(\begin{matrix} n \\ 2 \end{matrix})}{\frac{1}{2}\left[ \sum_{i} \left( \begin{matrix} a_{i} \\ 2 \end{matrix} \right)+\sum_{j} \left( \begin{matrix} b_{j} \\ 2 \end{matrix} \right) \right]-\left[ \sum_{i} \left( \begin{matrix} a_{i} \\ 2 \end{matrix} \right)\sum_{j} \left( \begin{matrix} b_{j} \\ 2 \end{matrix} \right) \right]/(\begin{matrix} n \\ 2 \end{matrix})}$ (1)

where $n_{ij}$ is the number of elements in both cluster $i$ in one cluster and cluster $j$ in the other cluster, $a_{i}$ is the sum of the elements in cluster $i$, and $b_{j}$ is the sum of the elements in cluster $j$. The denominator normalizes the index by accounting for the expected similarity of all pairwise combinations.

This metric provides a robust evaluation of clustering algorithms, ensuring that the results are both accurate and statistically significant. By incorporating ARI and NRI into the GOLDEN fusion framework, we achieve a thorough assessment of clustering methods across synthetic and benchmark datasets (gene ontology annotations)[,](#_bookmark1) leading to more reliable and interpretable analyses.

# The Clustering Algorithms Used for Density-Based Method

K-means clustering partitions a dataset into K clusters by minimizing within-cluster variance. The algorithm proceeds as follows:

- 1. Initialize $K$ cluster centroids randomly.
  2. Assign each data point $x_{i}$ to the nearest cluster centroid $c_{j}$ :

$\mathrm{argmin}_{j}\left\| x_{i}-c_{j} \right\|^{2}$ (2)

1. Update the cluster centroids based on the mean of the assigned points:

$c_{j}=\frac{1}{\left| C_{i} \right|}\sum_{x_{i}\in C_{j}} x_{i}$ (3)

1. Repeat steps 2 and 3 until convergence.

Agglomerative clustering is a hierarchical clustering method that builds nested clusters by successively merging or splitting them. The algorithm operates as follows:

1. Initialize each data point as its own cluster.
2. Compute the distance between all pairs of clusters using a linkage criterion (e.g., single, complete, average linkage):

${d(C_{i},C_{j})}_{j}=\mathrm{linkage}(C_{i},C_{j})$ (4)

1. Merge the pair of clusters with the smallest distance.
2. Repeat step 2 until the desired number of clusters is obtained.
3. Common linkage criteria include:

– Single linkage (minimum distance):

${d(C_{i},C_{j})}_{j}=\min_{x\in C_{i},y\in C_{j}} \left\| x-y \right\|$ (5)

– Complete linkage (maximum distance):

${d(C_{i},C_{j})}_{j}=\max_{x\in C_{i},y\in C_{j}} \left\| x-y \right\|$ (6)

– Average linkage:

${d(C_{i},C_{j})}_{j}=\frac{1}{\left| C_{i} \right|\left| C_{j} \right|}\sum_{x\in C_{i}} \sum_{y\in C_{j}} \left\| x-y \right\|$ (7)

HDBSCAN (Hierarchical Density-Based Spatial Clustering of Applications with Noise) is an extension of DBSCAN that converts it into a hierarchical clustering algorithm and then extracts a flat clustering based on the stability of clusters. The algorithm operates as follows:

1. Compute the mutual reachability distance between all pairs of points:

$d_{mreach}(x_{i},x_{j})=\max\left( \mathrm{cor}e_{k}\left( x_{i} \right),\mathrm{cor}e_{k}\left( x_{j} \right),d(x_{i},x_{j}) \right)$ (8)

where $\mathrm{cor}e_{k}\left( x \right)$ is the core distance of $x$, defined as the distance to its *k*-th nearest neighbor.

1. Construct the minimum spanning tree (MST) of the mutual reachability distances.
2. Condense the MST into a hierarchy of clusters based on the minimum cluster size.
3. Extract the flat clustering by selecting the most stable clusters from the hierarchy.

The Girvan-Newman algorithm detects communities by iteratively removing the edge with the highest betweenness centrality until the graph is divided into disconnected components. The betweenness centrality $C_{B}(e)$ of an edge e is defined as:

$C_{B}(e)=\sum_{s\neq t\neq e} \frac{\sigma\left( s,t | e \right)}{\sigma\left( s,t \right)}$ (9)

where $\sigma\left( s,t \right)$ is the total number of shortest paths from node $s$ to node $t$, and $\sigma\left( s,t | e \right)$ is the number of those paths that pass through edge $e$.

The Louvain algorithm is an iterative method that optimizes the modularity of the partition of the graph. Modularity $Q$ is defined as:

$Q=\frac{1}{2m}\sum_{i,j} \left[ A_{ij}-\frac{k_{i}k_{j}}{2m} \right]\delta\left( c_{i},c_{j} \right)$ (10)

where $A_{ij}$ is the adjacency matrix of the graph, $k_{i}$ and $k_{j}$ are the degrees of nodes $i$ and $j$, m is the number of edges, and $\delta\left( c_{i},c_{j} \right)$ is 1 if nodes $i$ and $j$ are in the same community, and 0 otherwise.

Spectral clustering uses the eigenvalues of the Laplacian matrix L of the graph to perform dimensionality reduction before applying k-means clustering. The Laplacian matrix is defined as:

$L=D-A$ (11)

where $D$ is the degree matrix and $A$ is the adjacency matrix. The algorithm proceeds by computing the first$k$ eigenvectors of $L$ (corresponding to the $k$ smallest eigenvalues) and using them as the new representation of the nodes. These representations are then clustered using k-means.


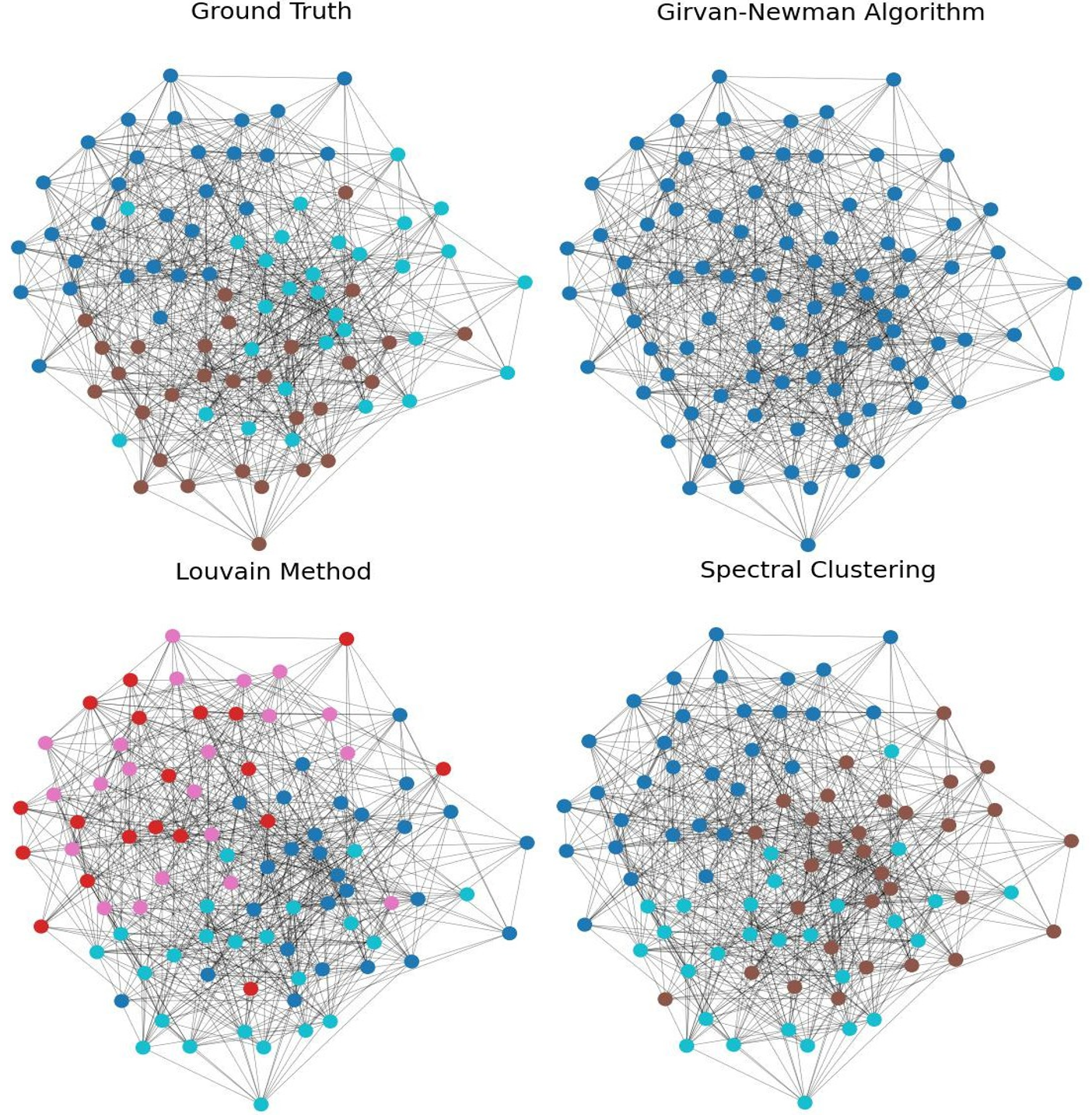


**Figure S1.** Visualization of Ground Truth and Clustering Results in SBM Network (Intra-Probability = 0.1, Inter-Probability = 0.3). This figure compares the ground truth clusters of the synthetic SBM network with clustering results from three algorithms: Girvan-Newman, Louvain, and Spectral Clustering. Spectral Clustering shows the closest match to the ground truth, highlighting its effectiveness in accurately identifying network structures under the given probability settings.

# Advanced network modeling using degree-corrected stochastic block models (DC-SBM) for training to generate CDI-$\beta$

The CDI is the calibrated output of supervised binary classifier that predicts whether a given GO network contains genuine substructure in clustering. Training data are generated synthetically using a Degree-Corrected Stochastic Block Model (DC-SBM) to derive the CDI-$\beta$ score: positive examples are graphs with k ∈ [2, 20] communities, where intra-cluster edge probability pin ∈ [0.30, 0.90] and inter-cluster probability pout < pin/2, cluster sizes are drawn from a Dirichlet distribution to represent both balanced and skewed partitions, and each cluster is augmented with a hub node; negative examples are Erdős–Rényi random graphs matched in average density so the model cannot distinguish clusterability by density alone. Both classes are further corrupted with 10–30% random edge rewiring to model real-world noise. From each graph, seven features are extracted: mean and variance of the local clustering coefficient, global transitivity, edge density, the first and second Laplacian spectral gaps (λ1 and λ2), and the modularity and community count returned by the Louvain algorithm. Four classifiers (Logistic Regression, Random Forest, XGBoost, and MLP) are trained on 800 such graphs and evaluated in three k-range buckets (small k ≤ 5, medium 6 ≤ k ≤ 12, large k > 12); the model with the highest mean F1 across buckets is retained. At inference time, the same seven features are extracted from the subgraph induced by a candidate set of GO terms and returns the classifier’s estimated probability of clusterability as the CDI-$\beta$.

# Applying Consensus Clustering to GOLDEN fusion for Optimal Cluster Number Selection

In order to enhance the robustness and accuracy of GOLDEN fusion’s clustering outputs, we integrated a consensus clustering algorithm into its workflow. Consensus clustering is a methodology designed for class discovery and clustering validation, particularly suited for analyzing complex biological data such as gene expression profiles. By aggregating the results of multiple runs of a clustering algorithm, consensus clustering provides a stable and reliable assessment of the clustering solution, mitigating the sensitivity to initial conditions that often affect traditional clustering methods.

For GOLDEN fusion, the consensus clustering algorithm was applied to the ensemble embedding matrix $D_{e}$, which combines topology-based and density-based information. By running various clustering algorithms, such as K-means and model-based Bayesian clustering, multiple times with random restarts, consensus clustering allowed us to identify the most stable and meaningful clusters. This approach not only helps in determining the optimal number of clusters but also provides insights into cluster membership stability and boundaries.


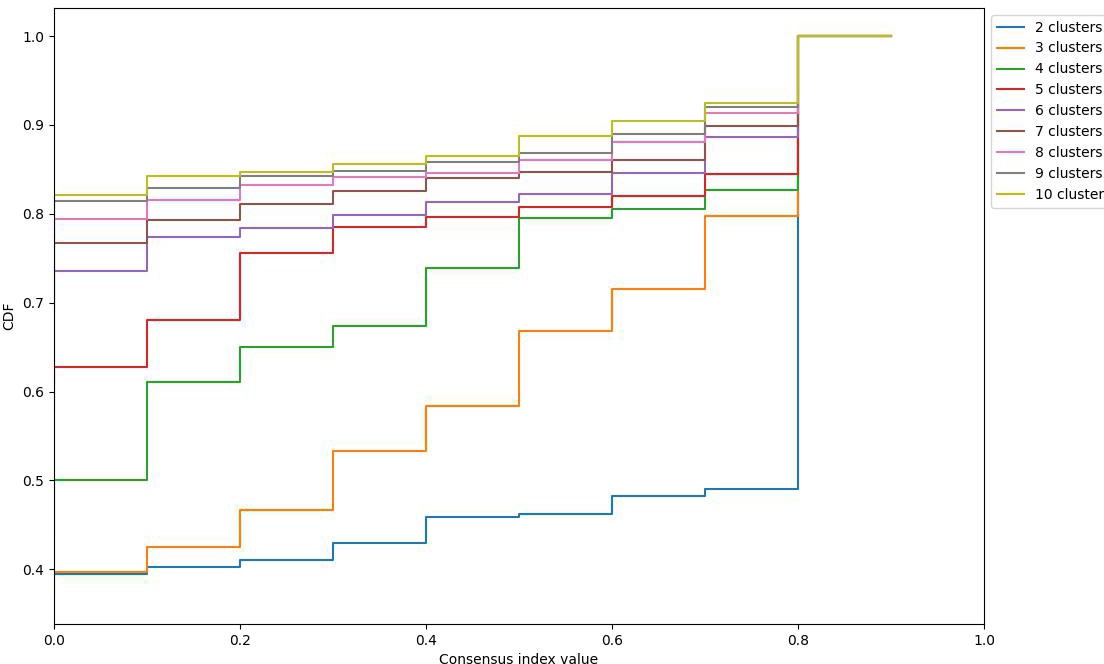
The application of consensus clustering to GOLDEN fusion ensures that the final putative super-PAGs are biologically meaningful and statistically robust. It also facilitates the visualization and interpretation of clustering results, providing researchers with a powerful tool to explore abstract biological concepts that integrate both bio-semantic and biological network information. By leveraging consensus clustering, GOLDEN fusion is better equipped to propose the best cluster number for a given set of genes or PAGs, thereby enhancing the overall utility and reliability of the method in functional genomics analysis. (see **Fig. S**[**2**](#_bookmark4))

**Figure S2**. CDF Plots from Consensus Clustering to Determine Optimal Cluster Number. It presents the Cumulative Distribution Function (CDF) plots corresponding to consensus matrices generated for different numbers of clusters (K = 2, 3, ..., 10). The analysis indicates that the best number of clusters is 2, as suggested by the CDF plot with the most stable and highest consensus index.

# The Process of Converting the Description into the Embeddings

The process works as follows:

1. **Input Sentences**: Given a description $d_{i}$ for a GOA term with an index $i$, SBERT processes the text to generate an embedding vector.
2. **Encoding**: SBERT encodes each description $d_{i}$ using the BERT model to produce a dense vector representation $u_{i}$:

$u_{i}=\mathrm{BERT}(d_{i})$ (12)

1. **Siamese Network Structure**: SBERT uses a Siamese network structure, where two identical BERT networks encode a pair of sentences (descriptions) independently. The outputs are then used to compute similarity.
2. **Triplet Network Structure**: For training, SBERT uses triplet loss, which ensures that the embedding of a description is closer to its positive pair (similar description) than to a negative pair (dissimilar description) by a margin. The triplet loss $L$ is defined as:

$L=max(0,\left\| u_{i}-u_{p} \right\|^{2}-\left\| u_{i}-u_{n} \right\|^{2}+\alpha)$ (13)

where $u_{i}$ is the anchor, $u_{p}$ is the positive example, $u_{n}$ is the negative example, and $\alpha$ is the margin.

1. **Cosine Similarity**: The resulting embeddings are compared using cosine similarity, which is given by:

$sim\left( u_{i},u_{j} \right)=\frac{u_{i}\cdot u_{j}}{\left\| u_{i} \right\|\left\| u_{j} \right\|}$ (14)

where $u_{i}$ and $u_{j}$ are the embedding vectors of two GOA term descriptions.

# The prompt of LLM-generated Summarization for Super-PAGs

The prompt we used for the summarization module is “You are a bioinformatics expert specializing in Gene Ontology annotation. When given a list of GO biological process descriptions, respond with exactly one concise sentence (30 words or fewer) that names the shared biological theme."
